# Supplementary material for: Putative Internal Control Genes in Bovine Milk Small Extracellular Vesicles Suitable for Normalization in Quantitative Real Time-Polymerase Chain Reaction
Source: Membranes (Basel). 2021 Nov 26;11(12):933. doi: 10.3390/membranes11120933 (PMC8709374; doi:10.3390/membranes11120933)
Supplement: Supplementary file 1 [file membranes-11-00933-s001.zip › membranes-1414001-supplementary.pdf]

Article

Putative Internal Control Genes in Bovine Milk Small Extracellular Vesicles Suitable for Normalization in Quantitative Real Time-Polymerase Chain Reaction

Md. Matiur Rahman [1,2,3], Shigeo Takashima [4], Yuji O. Kamatari [5], Yassien Badr [2,6], Kaori Shimizu [2], Ayaka Okada [2,7] and Yasuo Inoshima [1,2,7,8*]

1 The United Graduate School of Veterinary Sciences, Gifu University, 1-1 Yanagido, Gifu 501-1193, Japan; matiur.vetmed@gmail.com
2 Laboratory of Food and Environmental Hygiene, Cooperative Department of Veterinary Medicine, Gifu University, 1-1 Yanagido, Gifu 501-1193, Japan; yassienbadr1@gmail.com (Y.B.); skaori@gifu-u.ac.jp (K.S.); okadaa@gifu-u.ac.jp (A.O.)
3 Department of Medicine, Faculty of Veterinary, Animal and Biomedical Sciences, Sylhet Agricultural University, Sylhet-3100, Bangladesh
4 Division of Genomics Research, Life Science Research Center, Gifu University, 1-1 Yanagido, Gifu 501-1193, Japan; staka@gifu-u.ac.jp
5 Division of Instrumental Analysis, Life Science Research Center, Gifu University, 1-1 Yanagido, Gifu 501-1193, Japan; kamatari@gifu-u.ac.jp
6 Department of Animal Medicine, Faculty of Veterinary Medicine, Damanhour University, El-Beheira-22511, Egypt
7 Education and Research Center for Food Animal Health, Gifu University (GeFAH), 1-1 Yanagido, Gifu 501-1193, Japan
8 Joint Graduate School of Veterinary Sciences, Gifu University, 1-1 Yanagido, Gifu 501-1193, Japan
* Correspondence: inoshima@gifu-u.ac.jp

Supplementary Material Table S1. Primer sequences of nine candidate putative internal control genes in milk sEVs that were not detected in qRT-PCR.
sEVs, small extracellular vesicles; qRT-PCR, quantitative real time-polymerase chain reaction.

Name of the gene	Gene symbol	Primer sequence	References
Glyceraldehyde 3-phosphate dehydrogenase	GAPDH	F: 5'-CCGGGAAACTGTGGCGTGATGG-3'<br>R: 5'-AGGTGGAGGAGTGGGTGTCGCTGTT-3'	This study
Nuclear valosin-containing protein-like	NVL	F: 5'-TCTGCTTTGGTACGGGAGGC-3'<br>R: 5'-ACTCCAGCACCATCACTGCAT-3'	This study
Beta-2-microglobulin	B2M	F: 5'-AGCGTACTCCAAAGATTCAGGTT-3'<br>R: 5'-ATGATGCTGCTTACATGTCTCGAT-3'	This study
Serum albumin	ALB	F: 5'-TGCCCTGTGCAGAAGACTATCTA-3'<br>R: 5'-CGAGCTCAACAAGTGCAGTT-3'	This study
Antizyme inhibitor 1	OAZI	F: 5'-GCCAAACGCATTAACTGGCG-3'<br>R: 5'-TGTCCTCGCGGTTCTTGTG-3'	This study
TATA-box binding protein	TBP	F: 5'-TGAACGTCATGGATCAGAACAACA-3'<br>R: 5'-TGCCGTAAGGCATCATTGGA-3'	This study
Protein kinase CGMP-dependent 1	PRKG1	F: 5'-AGCACAAATGGTTTGAGGGCTTTA-3'<br>R: 5'-AGGTGGCGGTTCATCATTGTC-3'	This study
18S ribosomal RNA	18SrRNA	F: 5'-TTCGATGGTAGTCGCTGTGC-3'<br>R: 5'-TTGGATGTGGTAGCCGTTTCT-3'	[23]
28S ribosomal RNA	28SrRNA	F: 5'-CTAGTAACGGCGAGCGAAGA-3'<br>R: 5'-AGGCGTTCAGTCATTATCCAA-3'	[24]
